# Prospective motion correction for diffusion weighted EPI of the brain using an optical markerless tracker

**DOI:** 10.1002/mrm.28524

**Published:** 2020-09-29

**Authors:** Johan Berglund, Adam van Niekerk, Henric Rydén, Tim Sprenger, Enrico Avventi, Ola Norbeck, Stefan L. Glimberg, Oline V. Olesen, Stefan Skare

**Affiliations:** ^1^ Department of Clinical Neuroscience Karolinska Institutet Stockholm Sweden; ^2^ Department of Neuroradiology Karolinska University Hospital Stockholm Sweden; ^3^ MR Applied Science Laboratory GE Healthcare Stockholm Sweden; ^4^ TracInnovations Ballerup Denmark

**Keywords:** diffusion tensor imaging, diffusion weighted MRI, echo‐planar imaging, markerless motion tracking, real‐time movement correction

## Abstract

**Purpose:**

To enable motion‐robust diffusion weighted imaging of the brain using well‐established imaging techniques.

**Methods:**

An optical markerless tracking system was used to estimate and correct for rigid body motion of the head in real time during scanning. The imaging coordinate system was updated before each excitation pulse in a single‐shot EPI sequence accelerated by GRAPPA with motion‐robust calibration. Full Fourier imaging was used to reduce effects of motion during diffusion encoding. Subjects were imaged while performing prescribed motion patterns, each repeated with prospective motion correction on and off.

**Results:**

Prospective motion correction with dynamic ghost correction enabled high quality DWI in the presence of fast and continuous motion within a 10° range. Images acquired without motion were not degraded by the prospective correction. Calculated diffusion tensors tolerated the motion well, but ADC values were slightly increased.

**Conclusions:**

Prospective correction by markerless optical tracking minimizes patient interaction and appears to be well suited for EPI‐based DWI of patient groups unable to remain still including those who are not compliant with markers.

## 1 INTRODUCTION

In theory, arbitrary rigid body motion can be perfectly compensated for by adjusting the imaging coordinate system in real‐time, provided motion estimates of sufficiently high frequency, high precision, and low latency.^1^ Nonetheless, the same motion can invalidate the image if left uncorrected. Patient motion is typically some orders of magnitude faster than MR image acquisition, rendering the modality inherently susceptible to motion.^2^ Since each k‐space sample contributes to all voxels, motion during a small fraction of the image acquisition time may potentially corrupt the entire image. Due to this sensitivity, a substantial amount of MR images might be degraded by motion.^3^ The problems associated with motion yield impaired diagnostic quality, prolonged examination times due to repeated acquisitions, and may require anesthesia of patients incapable of lying still, such as children.

Diffusion weighted imaging (DWI) provides unique information about tissue microstructure, being sensitive to the magnitude and direction of molecular self‐diffusion. DWI is an essential component of many standard brain MR examinations and is the gold standard technique for the detection of stroke. In addition to the primary effect of moving the anatomy under study between consecutive readouts, a secondary effect is caused by motion during the diffusion sensitizing gradients. In particular, rotational motion during diffusion encoding will cause a translation in k‐space perpendicular to the diffusion direction and the rotational axis.^4^ This causes signal loss or even total signal dropout if the k‐space sampling matrix misses the center peak.^5^ Partial Fourier acquisition aggravates the problem, as the k‐space edge is shifted closer to center on the undersampled side.^6^ A third effect is produced by changes in susceptibility‐induced off‐resonances, extending beyond the source of the motion.^7^ This effect is of note in EPI‐based imaging, because off‐resonances cause significant spatial distortion due to the slow k‐space traversal in the phase encoding direction.

Favorably, brain imaging during motion is well‐posed for correction, since the rigid body assumption is acceptable, which simplifies pose estimation to six degrees of freedom.^8^ Retrospective correction^9^, ^10^ can be used to weed motion artifacts to some extent, but prospective correction techniques have the potential of nipping the motion in the bud. Single‐shot EPI is fairly robust to in‐plane motion, since a complete slice can be acquired on the order of 100 ms, which effectively “freezes” typical head motion.^11^ In principle, such motion can be corrected retrospectively, as opposed to through‐plane motion.^12^ Prospective correction techniques are, therefore, attractive to improve the resistance to through‐plane motion between slices and between volumes, as well as adverse spin‐history effects,^13^ for EPI‐based applications such as DWI,^14^ and functional MRI.^15^

The most convenient prospective correction approach is to use the MR system for internal motion navigation.^16^ This has the advantage of low cost and easy workflow, but may interfere with imaging and prolong sequence duration. Internal navigators introduce a conflict between the need for high precision estimates with high frame rates on one hand and keeping the scan time short on the other.^16^, ^17^, ^18^, ^19^

Prospective correction can be achieved without impairing pulse sequences, through optical tracking of markers attached to the subject.^14^, ^15^, ^20^, ^21^, ^22^, ^23^ This enables high precision tracking without prolonging the scan time, but raises requirements on subject compliance. Unfortunately, there may be a substantial overlap between patients non‐compliant with markers and those prone to move during imaging, including small children. Markers also increase subject preparation time and may have adverse effects due to marker detachment, slippage, or loss of visual contact.^24^, ^25^ Since optical tracking is independent of the MR system, some type of cross‐calibration is required between the tracking and MR systems that aligns their frames of reference.

Maclaren et al. compiled three goals for an ideal prospective motion correction method: high accuracy and precision, no patient interaction, and sequence independence.^26^ These requirements may be met by optical markerless motion tracking. The absence of additional patient interaction make markerless systems attractive in the clinical setting. Markerless head tracking uses the subject’s facial features as a reference. With the correct protocol, it is possible to extract these same features from an MR image, simplifying cross‐calibration. Prospective motion correction using a novel markerless motion tracker has been demonstrated for 3D gradient echo,^27^, ^28^ 3D fast spin echo,^28^ and 2D fast spin echo and inversion recovery sequences.^29^

In this work, we investigate the utility of optical markerless motion tracking to prospectively correct DWI of the brain using well‐established imaging techniques. To increase robustness against motion during diffusion encoding, we use single‐shot EPI with full Fourier acquisition. Sensitivity to geometric distortions is decreased using GRAPPA acceleration, with a short motion‐robust GRAPPA calibration volume acquired at the beginning of the scan. The technique is evaluated on volunteers performing different motion patterns with and without correction, and the effect of correction on images without motion is investigated.

## 2 METHODS

All imaging was performed on a 3T MR system (Signa Premier, GE Healthcare, Milwaukee, WI) using a 48‐channel head coil from the same vendor. Motion estimates for prospective correction were obtained with a markerless motion tracker (Tracoline TCL3.1m, research version provided by TracInnovations, Ballerup, Denmark), including the software TracSuite v3.0m. Both the hardware and software of the tracking system were customized to fit the current research project. Acquisitions were made in vivo in accordance with the institutional review board policy, and informed consent was obtained from both volunteers.

### 2.1 Markerless motion tracker

The MR‐compatible tracking system was setup as described by Frost et al.,^28^ with the scanning probe fixed to the patient table. The system uses active stereo vision based on a structured infrared light technique to generate 3D point clouds of the subject’s face with a frame rate of about 30 fps. These point clouds are co‐registered to a reference point cloud to determine rigid body pose estimates. A UDP socket was used to send the estimates to the MR system, and to receive table position and imaging sequence information. On the MR system used, the patient table is automatically moved to align the center of the slice stack with the magnet isocenter. It was, therefore, necessary to dynamically update the transformation matrix according to the table position. This enabled table movement without the need to repeat system cross‐calibration.^30^

One cross calibration image volume was acquired for each subject. A coronal 3D spoiled gradient echo with a FOV of 176/100/220 mm was positioned to cover the subject’s face. Other imaging parameters were: voxel size 1.0 mm isotropic; TE/TR/FA 4.5 ms/7.4 ms/ $15^{\circ }$; BW ±50 kHz; *R* = 2; acquisition time 59 seconds. After online reconstruction, the calibration image volume was automatically transferred to the tracking system computer, where it triggered a pop‐up window for semi‐automatic registration of a reference point cloud to the surface of the subject’s face that was extracted from the image volume.

### 2.2 Pulse sequence

The diffusion EPI sequence used in all experiments was developed in house using the KS Foundation abstraction layer.^31^ Synchronous prospective updates of the imaging coordinate system were applied before each excitation pulse, such that the slice position and angulation, FOV, and diffusion gradient direction could follow the motion of the head. Within the KS Foundation framework, such updates are implemented in a custom function which adjusts the transmit frequency and receive frequency and phase for translation updates, and uses the vendor provided function for rotation updates.

**FIGURE 1:**
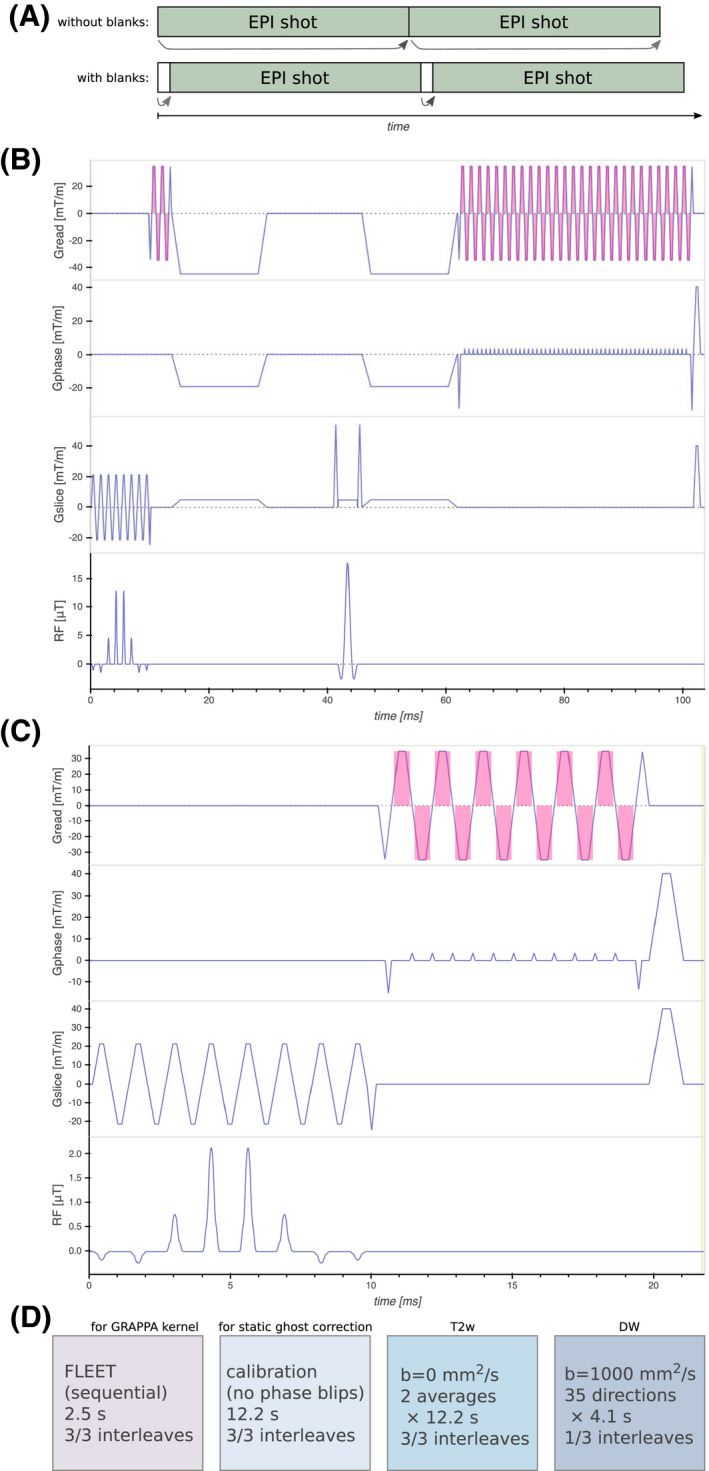
A: Since the prospective update of a shot is prepared during the preceding sequence playout, a 3 ms blank sequence was interleaved with the EPI sequence. This substantially reduced the additional latency of the updates. B: Pulse sequence diagram of the main EPI sequence. The spectral‐spatial excitation pulse followed by phase reference lines for dynamic ghost correction, Stejskal‐Tanner diffusion encoding, and EPI readout. The magenta shade indicates signal readout. C: Pulse sequence diagram of the FLEET sequence, which is essentially a stripped version of the main sequence. The same RF pulse but with lower flip angle is followed by a short EPI readout. D: Sequence of imaging volumes. For the first volume (FLEET), all shots for a slice were acquired sequentially, to enable motion‐robust GRAPPA calibration. The second calibration volume was acquired without phase encoding for static ghost correction (used to ghost correct the FLEET volume). The T2‐weighted volumes were fully shot but each shot was reconstructed with GRAPPA separately before averaging. The diffusion weighted volumes were single‐shot (*R* = 3). Prospective updates were applied once before the two calibration volumes and before each excitation for the imaging volumes. The total acquisition time was 3 minutes 1 second

The waveform preparation for a shot starts while the previous shot is played out. This introduces an additional update latency corresponding to the duration of a shot. To minimize this latency, a blank sequence was inserted between each EPI shot. A duration of 3 ms for the blank sequence was found sufficient to prepare the upcoming waveforms in time. This guarantees an additional latency no longer than 3 ms. The sequence timing is illustrated in Figure 1A.

Due to eddy currents and system time delays, misregistration between odd and even lines in the EPI train causes the infamous Nyquist ghost artifact. This is often corrected using a calibration volume, without phase encoding, as reference (denoted static ghost correction). Since the amount of misregistration is orientation dependent,^32^ static correction might not suffice when the slice orientation is updated.^1^ A short reference EPI readout without phase encoding was added after the excitation pulse. These navigator echoes enabled dynamic ghost correction. Stejskal‐Tanner diffusion encoding^33^ was used to achieve a short echo time and avoid errors from concomitant fields.^34^ The pulse sequence diagram is shown in Figure 1B.

For GRAPPA acceleration, a short calibration volume was acquired using the fast low‐angle excitation echo‐planar technique (FLEET).^35^ The FLEET pulse sequence diagram is shown in Figure 1C. Since all shots for a given slice are acquired sequentially, FLEET is exceptionally robust to motion. After the FLEET volume, a static ghost correction calibration volume was acquired using the imaging EPI sequence with the phase encoding gradients turned off. The two calibration volumes were not prospectively updated in order to allow static ghost correction, except for one update at the start of the acquisition. This avoided mismatch between calibration and prospectively corrected imaging volumes. The same was done for acquisitions without prospective correction, to get aligned slices over image series to facilitate comparison.

### 2.3 Reconstruction

Static ghost correction was applied to the FLEET volume before it was used for GRAPPA kernel estimation. Each shot in the imaging volumes was ghost corrected using its phase reference lines before GRAPPA reconstruction.^36^ All *b* = 0 s/mm^2^ shots were then averaged without co‐registration to form a single image volume. The diffusion weighted images were retrospectively aligned slice‐wise using non‐rigid registration, to compensate for eddy current distortion.^37^ The vendor provided technique PURE (Phased array Uniformity Enhancement) was used for receive coil inhomogeneity correction.

### 2.4 Experiments

In vivo DWI was performed using two *b* = 0 s/mm^2^ volumes each acquired in three shots, ie, k‐space consisted of three interleaves. For the diffusion weighted volumes, only one of the three interleaves was acquired. In other words, they were single shot with acceleration factor *R* = 3. Thirty‐five volumes were diffusion weighted (*b* = 1000 s/mm^2^) with uniformly distributed directions of the diffusion encoding. Thirty‐eight 4 mm axial slices with a 240 mm square FOV were acquired to cover the whole brain. The voxel size was 1.5 × 1.5 mm^2^, TR 4,058 ms, and ramp sampling was performed with a sampling BW of ±250 kHz. To improve robustness against rotation during diffusion encoding, full Fourier acquisition was employed, resulting in a TE of 77 ms. The time between excitations was 106.8 ms, resulting in an effective prospective update rate of almost 10 fps. The FLEET volume had a FA of $15^{\circ }$ and a voxel size of 1.5 × 6.7 mm. The three shots were acquired in 65 ms per slice, resulting in 2.5 s for the whole volume. The total acquisition time was 3 minutes 1 second. The ordered image volumes are shown in Figure 1D.

Videos of moving cross‐hairs were generated for the motion experiments, each corresponding to a different motion pattern. The videos were displayed on an fMRI screen placed behind the scanner bore and visible through a mirror mounted on the head coil. The subjects were instructed to follow the cross‐hairs with their nose, the purpose being to improve repeatability of the motion patterns.

To evaluate the effect of partial Fourier imaging, one subject was scanned with prospective motion correction (PMC) while performing the *circular* motion (described below). The hypothesis was that problems related to loss of diffusion weighted signal would increase with decreasing partial Fourier factor (PFF). Images with varying PFF were reconstructed using POCS,^38^ by discarding data retrospectively. One additional acquisition was made without voluntary motion as a reference.

A second subject was scanned while performing five different motion patterns, each repeated with PMC on/off and guided by videos of moving cross‐hairs. The instructions given to the subject were denoted as:

1. *Still*—Remain as still as possible;
2. *Stepwise*—Take one second to move to a new pseudo‐random position every six seconds;
3. *Yaw*—Move continuously center‐left‐right‐center as in “no.” Repeat every 12 seconds;
4. *Pitch*—Move continuously center‐up‐down‐center as in “yes.” Repeat every 15 seconds;
5. *Circular*—Move along a circle at constant speed with one cycle per 10 seconds.

To examine the need for dynamic ghost correction, the *still* and *stepwise* acquisitions were reconstructed with both static and dynamic ghost correction.

To investigate the impact of motion during GRAPPA calibration, the prospectively corrected *still* and *circular* acquisitions were reconstructed with GRAPPA calibration from either their corresponding *b* = 0 s/mm^2^ volume, the FLEET volume from the *still* scan, or the FLEET volume from the *circular* scan.

The impact of motion on ADC was analysed by calculating ADC histograms from the whole imaging volume and comparing it to a reference histogram without motion. In addition, histograms of the voxel‐by‐voxel ADC difference with respect to the reference were calculated.

To assess the impact of motion on principal diffusion direction and fractional anisotropy (FA), the color FA were manually inspected. Like in the ADC analysis, whole‐volume FA histograms and voxel‐by‐voxel FA difference histograms were calculated with respect to a reference.

## 3 RESULTS

For each subject, the time required for the calibration procedure was roughly 1 minutes 30 seconds: 10 seconds for positioning the volume; 1 min for data acquisition; 5–10 seconds for image reconstruction and data transfer; and 10–15 seconds for semi‐automatic registration.

**FIGURE 2:**
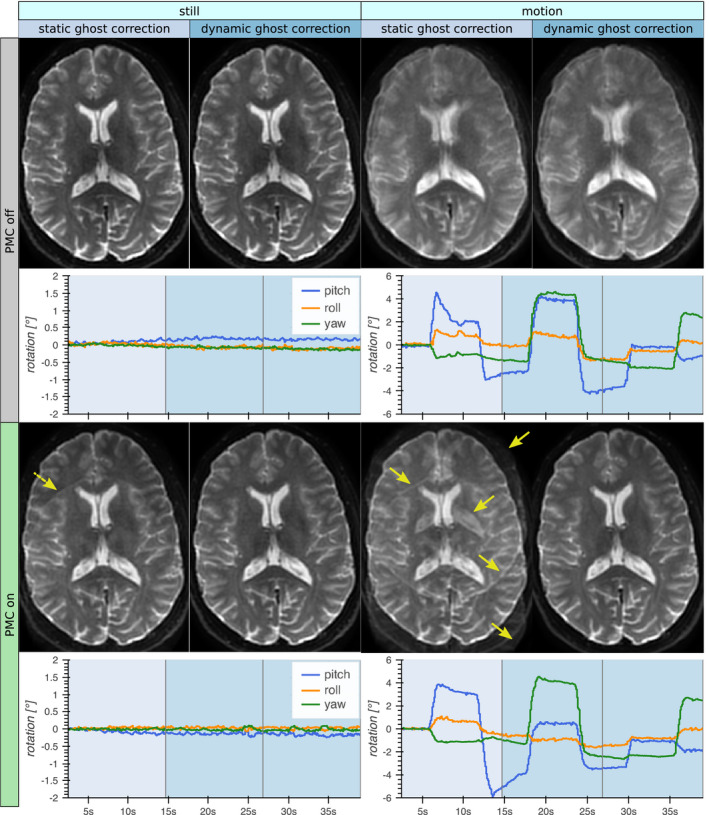
The *b* = 0 images from acquisitions with/without voluntary motion and with/without prospective motion correction (PMC). These datasets were reconstructed both with static ghost correction (using the ghost calibration volume) and dynamic ghost correction (using the phase reference lines). The rotational motion estimates for the calibration volume and the two *b* = 0 volumes are shown in the plots (note the differing scales on the *y*‐axes). Static ghost correction was successful without PMC, but even small updates of the FOV from involuntary motion issued ghost artifacts (dashed arrow). Larger motion resulted in severe ghost artifacts (solid arrows). Dynamic ghost correction enabled PMC without visible ghosting

The datasets that were reconstructed with both static and dynamic ghost correction are shown in Figure 2. Even for the acquisition without voluntary motion, the minor prospective updates resulted in slight ghosting for some slices when using static ghost correction. When using dynamic ghost correction, PMC did not increase the level of ghosting. The *still* dynamically corrected images with PMC were not inferior to the statically corrected images without PMC. Data corruption by motion was avoided to a large extent using PMC and dynamic ghost correction.

**FIGURE 3:**
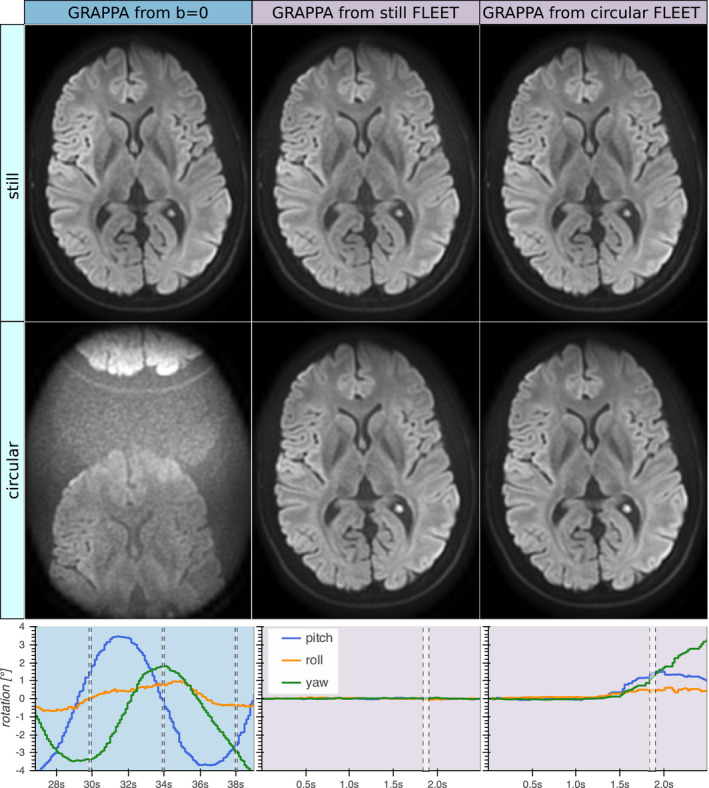
Prospectively motion corrected isoDWI acquired without and with voluntary motion (first and second rows, respectively). Both acquisitions were reconstructed using GRAPPA weights from either their *b* = 0 volume (left column), the FLEET volume from the acquisition without motion (middle column), or the FLEET volume from the acquisition with motion (right column). The plots show the rotational motion estimates from the *b* = 0 volume with motion (left), the FLEET volume without motion (middle), and the FLEET volume with motion (right). The timing for acquisition of the shown slice is indicated by the dashed vertical lines. Note that the three *b* = 0 shots are separated by *TR* = 4 seconds, while the FLEET shots were acquired sequentially, separated by 22 ms only. The *b* = 0 volume served well for GRAPPA calibration if no motion was present. However, extreme GRAPPA ghosts at thirds of the FOV were introduced by inter‐shot motion despite PMC and dynamic ghost correction, likely due to shot‐to‐shot variations of the coil sensitivities. The FLEET volumes worked well for GRAPPA calibration, even when motion was present during the FLEET acquisition

Prospectively corrected images reconstructed with various GRAPPA calibration data are shown in Figure 3. The *b* = 0 volume could be used successfully for GRAPPA calibration in the absence of voluntary motion, but yielded intolerable GRAPPA ghosts when the subject moved continuously. Using the FLEET volume for GRAPPA calibration did not deteriorate the image quality for the *still* acquisition, and it could also be used to reconstruct the motion dataset without notable ghosting. Even when performing GRAPPA calibration with a FLEET volume acquired during motion, equal image quality without significant ghosting was observed.

**FIGURE 4:**
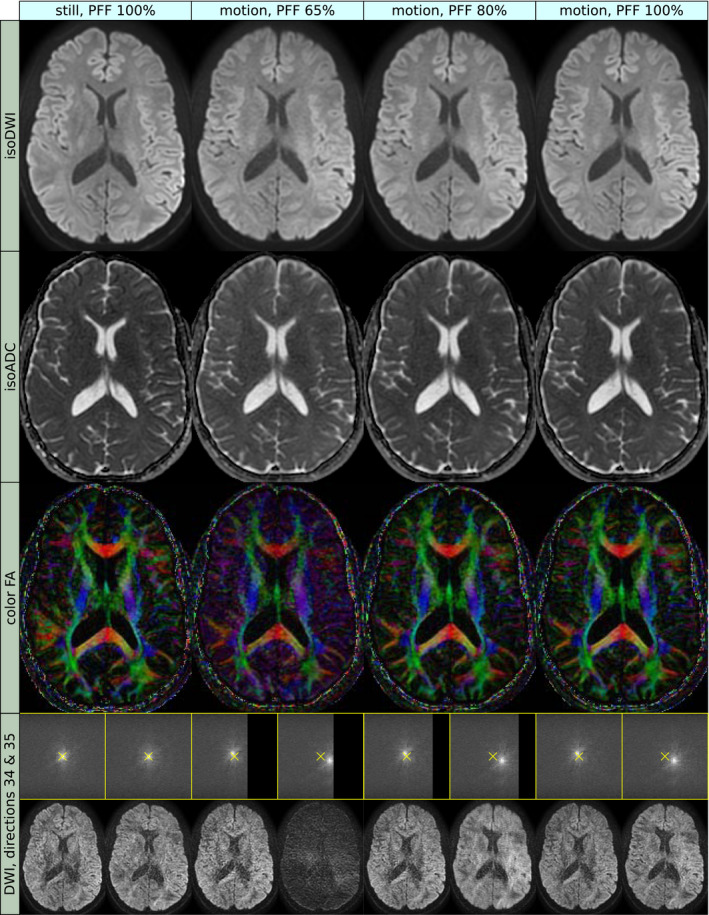
Prospectively corrected images were acquired during circular motion, and reconstructed with varying partial Fourier factor (PFF) (second to fourth column). Images without motion were acquired for reference (first column). The rows show isoDWI (first row), mean ADC (second row), Color Fractional Anisotropy (CFA, third row), and the k‐space and image space of the last two diffusion weighted volumes (bottom row). The images reconstructed with PFF 65% demonstrated loss of sharpness and contrast of the isoDWI, increased ADC values, and corrupt principal diffusion direction (as indicated by the color of the CFA), compared to the reference. The underlying FA (corresponding to intensity of the CFA) also appeared “flattened.” All these issues were mitigated with higher PFF. The observed effects are related to rotational motion during the diffusion encoding gradients, which induces a translation in k‐space perpendicular to the diffusion gradient and the axis of rotation. The shift will, therefore, vary between slices and diffusion directions. In the *still* acquisition, the k‐space center coincided with the center of the sampling matrix (yellow cross). In the acquisition with motion, some slices obtained a shift away from the undersampled part of k‐space and tolerated the partial Fourier undersampling well. Other slices were shifted toward the undersampled part, which resulted in blurring and signal loss to an increasing extent for lower PFF. Since the signal dropout effect is proportional to the b‐value, an erroneously increased ADC will result, and since the amount of signal dropout varies between diffusion directions, the principal diffusion direction might be altered

The acquisitions with varying PFF are shown in Figure 4. For high retrospective undersampling (PFF 65%), the motion induced unacceptable perturbation of the diffusion tensors. Conversely, full Fourier acquisition resulted in images that agreed well to the reference without motion, although the ADC appeared slightly increased.

**FIGURE 5:**
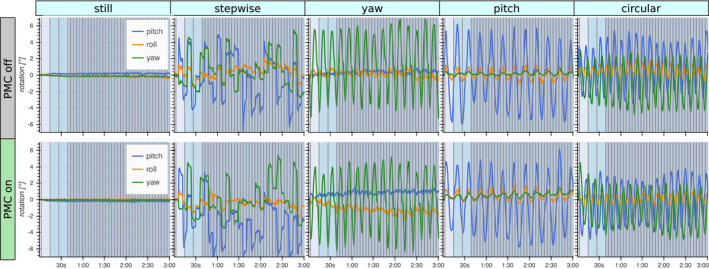
Estimated rotational motion during imaging of a volunteer performing five different motion patterns (columns). The acquisitions were repeated with PMC on and off. To achieve repeatable motion patterns, videos of moving cross‐hairs were displayed on a screen behind the bore that the subject could observe through a mirror. This resulted in highly synchronous motion between repeated acquisitions, but with slight variations in offset and amplitude. The performed motion had a range of about 10° rotation

The motion estimates from the five different motion patterns with PMC on and off are shown in Figure 5. The videos guiding the motion patterns resulted in very well synchronized motion between the prospectively corrected and uncorrected acquisitions. The absolute position and span of the motion was comparable but not identical.

**FIGURE 6:**
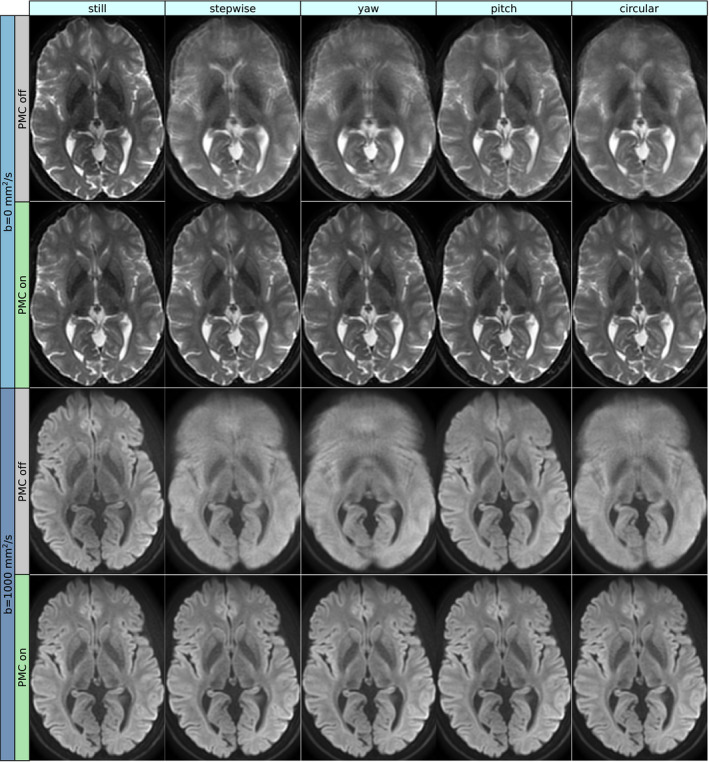
The *b* = 0 and isoDWI (*b* = 1000) images are shown for the five different motion patterns, acquired with and without PMC. Importantly, the images without voluntary motion were not degraded by PMC. The performed motion was large enough to severely deteriorate the image quality when not corrected for, but the prospectively corrected images were very faithful to the images without motion. The *pitch* motion had the most benign impact on the uncorrected images, but a distortion effect due to field inhomogeneity manifested in the front of the brain in the *b* = 0 image. In the uncorrected *b* = 1000 image, the effect was largely averaged out, but a loss of contrast remained. In the prospectively corrected images, this effect was absent

A representative slice of the *b* = 0 and *b* = 1000 images with and without PMC during the five motion patterns is shown in Figure 6. The performed motion patterns were extensive enough to severely corrupt the uncorrected images, especially in the frontal brain. The *pitch* motion was an exception because it had a much smaller impact, but the images were still significantly degraded. The image quality of the *still* images did not differ significantly when PMC was on or off. The prospectively corrected images were consistently of high quality, despite the presence of motion.

**FIGURE 7:**
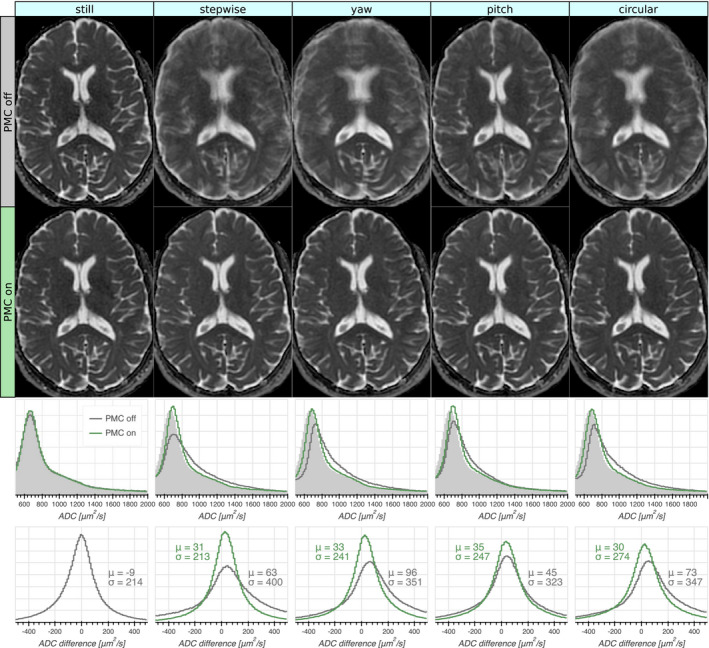
Mean ADC maps calculated from the acquisitions with varying motion patterns (columns) without and with PMC (first and second row, respectively). The third row shows whole‐volume ADC histograms with and without PMC, with the reference (prospectively corrected without motion) shaded in light grey. The PMC significantly improved the correspondence to the reference histogram. The bottom row shows whole‐volume histograms of the voxel‐wise ADC difference to the reference with the mean (*μ*) and standard deviation (*σ*) indicated. The prospectively corrected ADC maps had error profiles quite similar to those that were uncorrected without motion, but with slightly higher ADC values. The ADC maps without PMC had broader error profiles with a larger shift toward higher ADC:s

Calculated mean ADC maps for the five motion patterns with and without PMC are shown in Figure 7, together with ADC histograms and ADC difference histograms. Slightly higher values are visible in the ADC maps for the acquisitions with motion. This was verified by the histogram analysis, revealing a shift of the ADC mode of about +5% for the motion datasets. Otherwise, the prospectively corrected ADC histograms matched the *still* reference well, as opposed to the uncorrected data.

**FIGURE 8:**
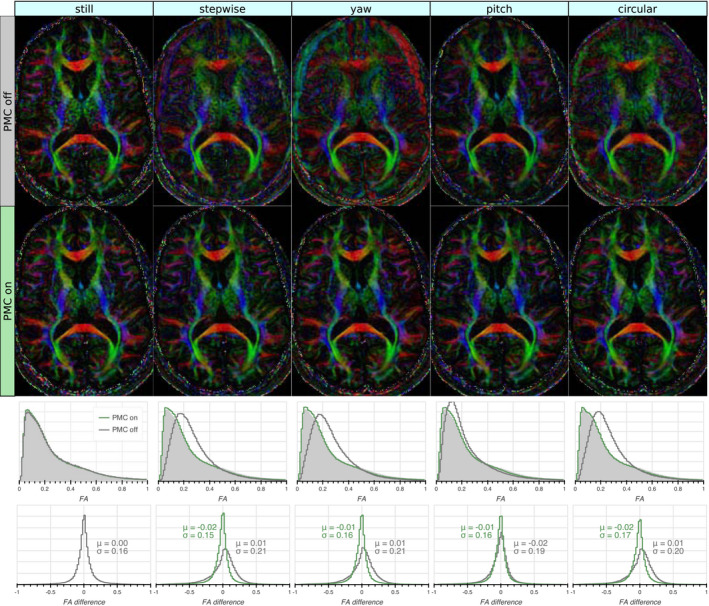
Color Fractional Anisotropy maps calculated from the acquisitions with varying motion patterns (columns) without and with PMC (first and second row, respectively). The uncorrected motion corrupted the CFA maps, while the CFA maps from the prospectively corrected acquisitions agreed well with those acquired without motion. The third row shows whole‐volume Fractional Anisotropy (FA) histograms with and without PMC, with the reference (prospectively corrected without motion) shaded in light grey. The FA histograms with PMC on were in close agreement with the reference, while those with PMC off were shifted toward higher values. The bottom row shows whole‐volume histograms of the voxel‐wise FA difference to the reference with the mean (*μ*) and standard deviation (*σ*) indicated. The error distributions of the FAs with PMC on were very similar to those without motion and PMC off. The FAs acquired without PMC had broader error distributions that were skewed toward higher FAs, although their mean was close to zero

The inspection of color FA maps indicated that the principal diffusion direction matched the *still* reference very well for all motion patterns with PMC. A representative slice is shown in Figure 8, together with the FA histograms and FA difference histograms. The histogram analysis showed that uncorrected motion resulted in a shift toward higher FA, while excellent agreement with the reference was seen for the prospectively corrected acquisitions.

## 4 DISCUSSION

This report has showed that EPI‐based DWI can be made robust to motion with the use of a markerless motion tracker and well‐established imaging techniques. It would be straightforward to combine these imaging techniques with alternative external methods for motion estimation, and comparable robustness to motion would be expected provided sufficient performance of the motion estimates. The imaging parameters were intended to reflect a typical clinical setting with a single low b‐value and a moderate resolution of 1.5 mm. Beyond prospective updates, the simple strategy to mitigate motion was to use single‐shot EPI with full Fourier acquisition. The experiments demonstrated high image quality despite the presence of substantial motion, but the limits were not explored regarding how much motion could be endured with acceptable image quality. Such limits are related to the update frequency, latency, and precision in the estimates. With the proposed method, the update frequency was limited by the once‐per‐excitation update strategy (∼10 fps), rather than by the frame rate of the tracking system (∼30 fps). According to previously reported phantom measurements, the tracking system has a latency on the order of 0.1 seconds and a precision of about 0.01 mm and 0.01°.^28^ Sufficient precision was demonstrated here by the non‐inferiority of the prospectively corrected images without voluntary motion.

A disadvantage of external tracking is the additional need for cross‐calibration between the tracking and imaging systems. In this work, one calibration image was acquired for each subject with 1 mm isotropic voxels in 59 seconds. According to our current experience, 2 mm isotropic voxels are sufficient, allowing a substantial reduction in the additional scan time needed for cross‐calibration. In essence, the cross‐calibration measures the position and orientation of the tracking probe relative to the magnet isocenter. Since the patient table position was taken into account, one calibration per setup of the tracking system would suffice. Although not explored here, the calibration could then be performed using an anthropomorphic phantom, to avoid the need of additional scan time for patients prone to move. Further, a permanently fixed tracking probe would allow a once‐off cross calibration.

The proposed sequence modifications come with some additional scan time; the FLEET volume required 2.5 seconds, the blank sequence added 5 seconds, and the phase reference lines added 2 seconds in total. This resulted in a combined scan time increase of about 5%. The phase reference lines also increased the minimum TE from 76 to 77 ms. Partial Fourier factors of 80%/65% would shorten the scan time by 11%/15%. Notwithstanding the prolonged acquisition, full Fourier imaging increases the fraction of scan time spent sampling the signal, which improves the SNR efficiency of the sequence.

In MRI, motion often results in ghosting due to signal differences between shots. While this can be avoided by advanced reconstruction techniques,^23^ we used the simpler approach of single‐shot EPI (even the fully shot *b* = 0 volumes were effectively single‐shot, since each shot was reconstructed separately using GRAPPA^36^).

A problem specific to DWI is the k‐space shift induced by rotational motion during the diffusion encoding gradients. The strategy employed in this work was to counter these detrimental effects by asserting full k‐space coverage for each shot. This helps avoid signal loss from in‐plane shifts, but not from through‐plane dephasing. Full k‐space coverage in the frequency encoding direction was accomplished using single‐shot EPI. Multi‐shot readout strategies, such as readout‐segmented EPI,^23^, ^39^ PROPELLER,^40^ and short axis PROPELLER EPI,^41^, ^42^ may be more susceptible to signal loss from in‐plane shifts. In the phase encoding direction, partial Fourier undersampling was omitted to maintain full k‐space coverage. The increased efficacy of prospective motion correction using full Fourier was demonstrated by the retrospective undersampling experiment (Figure 4). This padding against signal loss was gained at the expense of longer TE and scan time; a PFF of 80%/65% would shorten the TE from 77 ms to 66/62 ms. Partial Fourier imaging can be used with homodyne filters aligned to the shifted k‐space peaks.^6^ This reduces errors when the peak is shifted into the fully sampled half, but is ineffective in the opposite case, thus only solving half the problem. Another approach is to measure the induced shift, either with navigator echoes^43^ or indirectly using motion estimates,^44^ and shift the k‐space back using compensation blips that are updated in real time. Alternatively, the shift can be pre‐compensated using gradient moment nulling techniques.^45^ This mitigates effects from both translational and rotational motion, including through‐plane dephasing. Unfortunately, the TE becomes substantially increased^46^ without improved sampling efficiency, unlike full Fourier imaging. In theory, the ultimate strategy would be to update the imaging coordinate system continuously according to the motion. This would account for motion of any order. However, both compensation blips based on motion estimates^44^ and frequent updates during gradient playouts^22^ put high demands on latency and update rate of the tracking system. The implementation of updates during gradient playouts is also not trivial, as gradient slewing must be taken into account. Updating during waveform playouts was found infeasible given the software architecture of the MR system used in this work. Intra‐shot updates would require a modified ghost correction strategy, such as additional reference lines or correction based on imaging data.^23^, ^47^

The tolerance to k‐space shifts is not determined by the PFF per se, but rather by the extent of the central symmetrically sampled portion of k‐space. Therefore, higher resolution allows the use of lower PFF. The issue of through plane dephasing was not handled in this work. This was motivated by the more gradual signal loss as function of angular velocity, compared to the sudden signal drop of in‐plane dephasing.^44^ The resistance to through‐plane dephasing can be improved using thinner slices. This increases sensitivity to “nodding motion,” and is, therefore, well wedded with prospective motion correction.The moderate in‐plane resolution could be increased using higher acceleration factors, or at the cost of increased spatial distortions. Readout‐segmented EPI might be better suited if DWI of even higher resolution is desired.^39^

Despite prospective motion correction and full Fourier acquisition, a 5% increase in ADC persisted in the motion datasets, which was in line with previous reports.^19^ This could possibly be attributed to rotational motion during the diffusion encoding gradients. The resulting shifts in k‐space lead to some signal loss in the diffusion weighted images, but not in the *b* = 0 images, effectively introducing a positive bias of the ADC. Indeed, the ADC values increased with undersampling in the partial Fourier experiment (Figure 4). The signal loss from through‐plane dephasing was probably dominant due to the larger voxel size in this dimension. This bias is expected to increase for higher b‐values.

The FA tended to flatten out in the presence of uncorrected motion, i.e., a decrease in high‐FA tissue (white matter), and an increase in low‐FA tissue (gray matter, CSF). Overall, this led to a shift of the histogram mode toward higher FA (Figure 8). Dissimilar to the ADC values, this effect could be counteracted by prospective correction.

The diffusion encoding was implemented using standard Stejskal‐Tanner gradients.^33^ Considering the dead time between the first diffusion gradient and the refocusing pulse (Figure 1B), more advanced diffusion gradient waveforms^48^ could potentially be used to lower the TE at the expense of additional concomitant field dephasing.^34^ The distortions caused by the eddy currents from the relatively rapid slewing Stejskal‐Tanner gradients^49^ were successfully corrected retrospectively. The use of a large number of diffusion encoding directions was preferred over multiple averages of a small number of directions, in order to avoid orientation dependent variance of the diffusion tensor derived quantities.^50^, ^51^ Such orientation dependence may introduce bias and decrease reproducibility of diffusion measurements when motion is present.^52^ Diffusion encoding schemes with a small number of diffusion directions can take advantage of the combined amplitude of several physical gradient axes, allowing to shorten the TE.^51^ This is, however, incompatible with prospective updates of the diffusion gradients, since any given diffusion direction might become updated to coincide with a physical gradient axis.

A potential problem with motion during EPI‐based imaging is susceptibility field induced distortions. Since the susceptibility induced off‐resonance field varies with rotation, the distortions will change with orientation of the head beyond the domain of rigid body motion. Although the distortion effect was evident in the images, it did not appear worse in the motion datasets, except for some regions outside the brain, with a strong susceptibility gradient, such as the eyeballs. Thus, distortion correction^53^ was not considered necessary here, but may be crucial at higher resolution, at higher field strength, for lower acceleration factors, or for patients with metallic implants.

One effect of letting the FOV follow a moving subject is that the sensitivities of the stationary coil elements appear to move instead. This might lead to a mismatch between parallel imaging calibration data and imaging data, eventually causing artifacts. Despite a high number of coil elements and motion within a 10° range, such artifacts were not observed in these experiments.

The described motion correction strategies are applicable to any EPI‐based application, such as perfusion, arterial spin labeling, fMRI, and fast multi‐contrast sequences.^11^ The latter involve inversion pulses that may severely alter the magnetization history in the presence of motion. Such effects can be countered by prospective but not retrospective correction.^13^

Apart from the non‐rigid eddy current correction, no retrospective motion correction was performed in this work. Even though some additional improvement in image sharpness might be gained, retrospective correction techniques for DWI have been well explored elsewhere,^9^, ^10^, ^39^ and were left out here in order to limit the scope.

## 5 CONCLUSION

Markerless motion tracking can be used for prospective motion correction of standard EPI‐based diffusion MR. The precision and accuracy of the estimates were sufficient to maintain image quality of the prospectively corrected datasets without voluntary motion. Rapid and continuous motion within a 10° range was well tolerated by applying updates before each excitation pulse. The effects of orientation updates on the registration of odd and even k‐space lines were effectively accounted for by performing dynamic ghost correction. Using FLEET as a fast and motion‐robust calibration volume allowed regular GRAPPA reconstruction. Single‐shot full Fourier acquisition maintained the directional integrity of the diffusion tensors despite motion during the diffusion sensitizing gradients. ADC values were slightly increased in acquisitions with deliberate motion. Otherwise, the image quality of the prospectively corrected datasets acquired during motion were comparable to the reference without motion, even for motion patterns that severely degraded uncorrected images. The described technique appears suitable for clinical DWI of patients unable to remain still.

## CONFLICT OF INTEREST

Tim Sprenger is an employee of GE Healthcare. Stefan Glimberg and Oline Olesen are employees of TracInnovations. Research support was obtained from GE Healthcare.
